# Glucoside xylosyltransferase 2 as a diagnostic and prognostic marker in gastric cancer via comprehensive analysis

**DOI:** 10.1080/21655979.2021.1967067

**Published:** 2021-09-10

**Authors:** Yunxia Zhao, Shangshang Hu, Jinyan Zhang, Zhaogen Cai, Shuanhu Wang, Mulin Liu, Jing Dai, Yu Gao

**Affiliations:** a Department of Basic Medical College, Bengbu Medical College, Bengbu, China; b Research Center of Clinical Laboratory Science, School of Laboratory Medicine, Bengbu Medical College, Bengbu, China; c School of Life Science, Bengbu Medical College, Bengbu, China; d Anhui Province Key Laboratory of Translational Cancer Research, Bengbu Medical College, Bengbu, China; e Department of Pathology, First Affiliated Hospital of Bengbu Medical College, Bengbu Medical College, Bengbu, China; f Department of Gastrointestinal Surgery, The First Affiliated Hospital of Bengbu Medical College, Bengbu, Anhui, China

**Keywords:** Glucoside xylosyltransferase 2 (GXYLT2), gastric cancer (GC), prognosis, overall survival, immune cell infiltration, bioinformatics analysis

## Abstract

To investigate the potential role of GXYLT2 (glucoside xylosyltransferase 2) in gastric cancer (GC), the TCGA (The Cancer Genome Atlas) database and Gene Expression Omnibus (GEO) dataset were used to evaluate GXYLT2 mRNA expression, and the standardized mean difference and diagnostic value were comprehensively assessed. Survival analysis and univariate/multivariate cox regression analysis were performed to evaluate the prognostic value of GXYLT2 in GC patients. The correlation between GXYLT2 and tumor immune cells was identified by using the CIBERSORT algorithm. The results showed that GXYLT2 expression level was significantly increased in GC tissues. GXYLT2 expression was significantly correlated with the grade, stage, and invasion depth of gastric cancer. Overall survival was reduced in the high GXYLT2 expression group. Univariate and multivariate Cox regression analyses showed that GXYLT2 was a reliable prognostic factor. GSEA showed that GXYLT2 might participate in the development of GC through tumor-related pathways. The expression of GXYLT2 was positively correlated with 5 tumor-infiltrating immune cells (resting dendritic cells, m2 macrophages, monocytes, active NK cells and resting mast cells), and was negatively correlated with 6 tumor-infiltrating immune cells (plasma cells, activated memory CD4 T cells, resting NK cells, activated dendritic cells, and activated neutrophils and mast cells). Through cell experiment verification, GXYLT2 expression level in gastric cancer cells was found to be high, which verified the results from the bioinformatics analysis. Furthermore, immunohistochemical staining results also showed that GC tissues had positive GXYLT2 expression. In summary, GXYLT2 might be a potential diagnostic and prognostic biomarker for gastric cancer.

## Introduction

Gastric cancer (GC), the fifth most commonly diagnosed cancer worldwide, is the third leading cause of cancer death and remains a challenge in the field of oncology [1]. Although the incidence of GC has decreased in the past several decades, the prognosis for patients with advanced disease is still poor, with surveys showing that the 5-year survival rate for patients with advanced GC is approximately 10% [2,3]. GC is a disease with complex mechanisms involving multiple genetic alterations and abnormal gene expression [4]. Therefore, it is crucial to develop improved methods to diagnose GC early.

Glucoside xylosyltransferase 2 (GXYLT2), also known as glycosyltransferase 8 domain-containing protein 4 (GLT8D4), is located on chromosome 3p13 and encodes a protein with 443 amino acids and alpha-1,3-D-xylosyltransferase activity [5]. The enzyme has typical characteristics of a type II Golgi glycosyltransferase: a transmembrane region, a small N-terminal cytoplasmic structural domain and a C-terminal catalytic structural domain [6]. Previous reports showed that GXYLT2 could promote glycosyltransferase expression in human myeloid leukemia cells via Notch pathway activation, and it could also promote cell proliferation and migration by altering the Notch pathway in a variety of tumor cells [7], such as MDA-MB-231, BGC-823 and SGC-7901 cells [8]. BGC-823 was taken from the primary focus of gastric cancer, and SGC-7901 was taken from perigastric metastatic lymph nodes. BGC-823 and SGC-7901 had high lubricities and high transfer rates. Although GXYLT2 accelerates cell growth and migration in human cancer cells, to the best of our knowledge, there have been no reports on the role of GXYLT2 in the pathogenesis of gastric cancer.

In our pilot study, the expression levels of GXYLT2 were significantly different between GC tissue and nor normal gastric tissue by analyzing the data from the TCGA (The Cancer Genome Atlas) database and GEO (Gene Expression Omnibus) database. Hence, we hypothesized that GXYLT2 might be associated with GC progression and a prognostic marker of gastric cancer. The present study aimed to explore the value of GXYLT2 in assessing GC prognosis. The potential value of GXYLT2 in gastric cancer was comprehensively assessed, and the results were initially validated *in vitro* by performing qRT-PCR and immunohistochemistry assays.

## Materials and methods

### Data collection for GXYLT2 expression

The data used for GXYLT2 mRNA expression analysis included RNA sequencing data and microarray expression data. The RNA sequencing data for GC were obtained from the TCGA database (https://portal.gdc.cancer.gov/). The microarray data containing GC samples were acquired from the GEO database (3.https://www.ncbi.nlm.nih.gov/geo/) utilizing the following keywords: (stomach OR gastric) AND (cancer OR carcinoma OR tumor) specify the search results. The following exclusion criteria for GEO data were used: (1) datasets containing only GC tissues without normal gastric tissues, (2) datasets containing only animal samples or cell samples, and (3) datasets containing fewer than 10 samples in normal or tumor groups.

### Analysis of GXYLT2 mRNA expression levels in gastric cancer

For the standardized mean difference (SMD), Cohen’s classification was applied to estimate the overall effect size [9]. The results of SMD < 0.2 meant minor effects, SMD between 0.2 and 0.8 meant medium effects, and SMD > 0.8 meant major effects. When heterogeneity existed (*I^2^ > 50%*), the random effect model was selected for the datasets from the GEO and TCGA. The results were displayed in a forest plot, and publication bias was applied to assess the comprehensive quality. Continuous variables of GXYLT2 expression were transformed into counts of true positive (TP), false positive (FP), false negative (FP), and true negative (TN), and sensitivity (SEN), specificity (SPE), positive likelihood ratio (PLR), and negative likelihood ratio (NLR) and diagnostic odds ratio (DOR). The higher the PLR value (sensitivity/(1-specificity)), the higher the probability that the test result is truly positive. The smaller the NLR value ((1-sensitivity)/specificity), the more likely it is to be true negative when the test result is negative. The wide receiver operating characteristic curve (summary ROC, SROC) was mapped.

### Prognostic analysis of GC patients based on GXYLT2 expression levels

Among GC patients (n = 375) from the TCGA database, 344 clinical samples with survival time were selected for prognostic analysis. Survival curves regarding GXYLT2 expression levels were plotted using the R software survival package. Univariate/multivariate Cox analysis was performed to assess the prognostic effect of clinical variables on GC patients. The correlation of GXYLT2 expression with clinicopathological staging characteristics was evaluated using the R software Wilcoxon rank-sum test. 95% confidence intervals and hazard ratios (HRs) were calculated.

### Gene set enrichment analysis

The TCGA database transcriptome samples (n = 375) were downloaded and split into two groups (high expression group vs low expression group), depending on the median GXYLT2 expression level. GSEA_4.1.0 enrichment software was used for analysis, and the enrichment pathway was set to be significant for nominal (NOM) p < 0.05 and false discovery rate (FDR) q < 0.05 [10].

### Analysis of the correlation between GXYLT2 and tumor-infiltrating immune cells in gastric cancer

The proportion of immune cells and the number of infiltrates in all gastric cancer samples from the TCGA database were calculated using the e1071 package and the preprocessCore package of R software [11] and based on the CIBERSORT algorithm [12], and then samples were screened according to a P < 0.05. Correlations between immune cells were calculated according to the R software corrplot package, and the Pearson coefficient was used for significance testing. Immune cell differential analysis and immune cell correlation analysis were performed using R software, and finally, immune cells with significant GXYLT2 expression were derived by Venn diagram.

### Cell culture

Five cell lines, including four GC cell lines (HGC-27, MGC-803, BGC-823, and SGC-7901) and gastric epithelial GSE-1 cells, were cultured in DMEM (Gibco, Thermo Fisher Scientific, USA) with 10% fetal bovine serum (Life Technologies, USA) and 1% penicillin and streptomycin (100 U/mL penicillin and 100 μg/mL streptomycin, Life Technologies) at 37°C in a humidified incubator containing 5% CO_2_.

### RNA extraction and qRT‑PCR

Total RNAs were extracted from cells using TRIzol reagent (Invitrogen, CA, USA). TRIzol extraction is an effective method for isolating total RNAs, including small RNAs. In the process of extracting RNA by TRIzol method, chloroform was added into homogenized sample. The mixture was shaken vigorously. After centrifuged, the mixture was divided into water sample layer and organic layer, and RNA existed in water sample layer. After collecting the upper water sample, RNA could be obtained by isopropanol precipitation [13]. If not handled properly, TRIzol might be a health hazard because containing phenol and guanidine isothiocyanate. The contaminated genomic DNA was removed with RQ1 DNase (Promega, Madison, WI, USA) digestion, and the total RNA was directly amplified by TransScript Green One-Step qRT-PCR SuperMix (Transgen Biotechnology, Beijing, China). All qPCR reactions were performed in the Step One Plus Real-Time PCR System (Life Technologies). The primers used for GXYLT2 expression analysis were as following: the forward primer 5ʹ-TCTGAAGCCCGAGTTTGATAAGC-3ʹ and the reverse primer 5ʹ-TGATGGGGTAGATTCTGTGCT-3ʹ. GAPDH (glyceraldehyde-3-phosphate dehydrogenase) was used as an internal reference gene. The forward primer sequence was 5ʹ-ACAGCCTCAAGATCATCAGC-3ʹ, and the reverse primer sequence was 5ʹ-GGTCATGAGTCCTTCCACGAT-3ʹ.

### Immunohistochemistry analysis

Formalin-fixed and paraffin-embedded human gastric tissue specimens were used for immunohistochemical analysis. GC tissues were obtained from the First Affiliated Hospital of Bengbu Medical College. None of the patients received any radiotherapy or chemotherapy before surgery. A total of 15 patients with GC were enrolled in this study. A 1:400 dilution of anti-GXYLT2 (Bioss, China) was used as the primary antibody. Immunohistochemical analysis was conducted by two independent pathologists. The intensity was scored as 0 = negative, 1 = weak, 2 = moderate, and 3 = strong. The extent of positive cells less than 10% was scored as 1, 11%-50% was 2, 51% to 75% was 3, and >75% was 4. The final score was determined by multiplying the intensity and extent positivity scores (from 0 to 12). The scores ≥3 was considered positive. This study was approved by the Institutional Ethics Board of Bengbu Medical College (No. 2020LK238).

### Statistical analysis

Statistical analysis was performed using R version 4.0.3 software. Independent sample t-test was employed to analyze the differential expression levels of GXYLT2 in GC tissues and normal gastric tissues. The chi-square test was applied to analyze the link between the GXYLT2 expression levels and clinicopathological features of GC patients. The survival analysis was performed using Kaplan-Meier analysis. The Wilcoxon rank-sum test was applied for the analysis of the difference between tumor immune cells and GXYLT2 expression in gastric cancer, and the Pearson coefficient was used for the correlation between tumor immune cells and GXYLT2. The differences were statistically significant when P < 0.05.

## Results

In the current study, the expression profiles of GXYLT2 were significantly up-regulated in GC tissues by Comprehensive analysis using multiple databases. Based on TCGA data, the overall survival of GXYLT2 patients was significantly reduced in the GXYLT2 high expression group. Moreover, univariate cox regression analysis and multivariate cox regression analysis showed that GXYLT2 expression might be reliable prognostic indicators in GC patients. Enrichment analysis showed that GXYLT2 might participate in the development of GC through cell adhesion molecules and tumor-related pathways. Further, GXYLT2 expression was correlated with tumor-infiltrating immune cells in GC patients. Taken together, the results suggested that GXYLT2 might be used as a diagnostic and prognostic marker in gastric cancer.

### GXYLT2 was highly expressed in gastric cancer tissues based on TCGA and GEO databases

**Figure 1.**
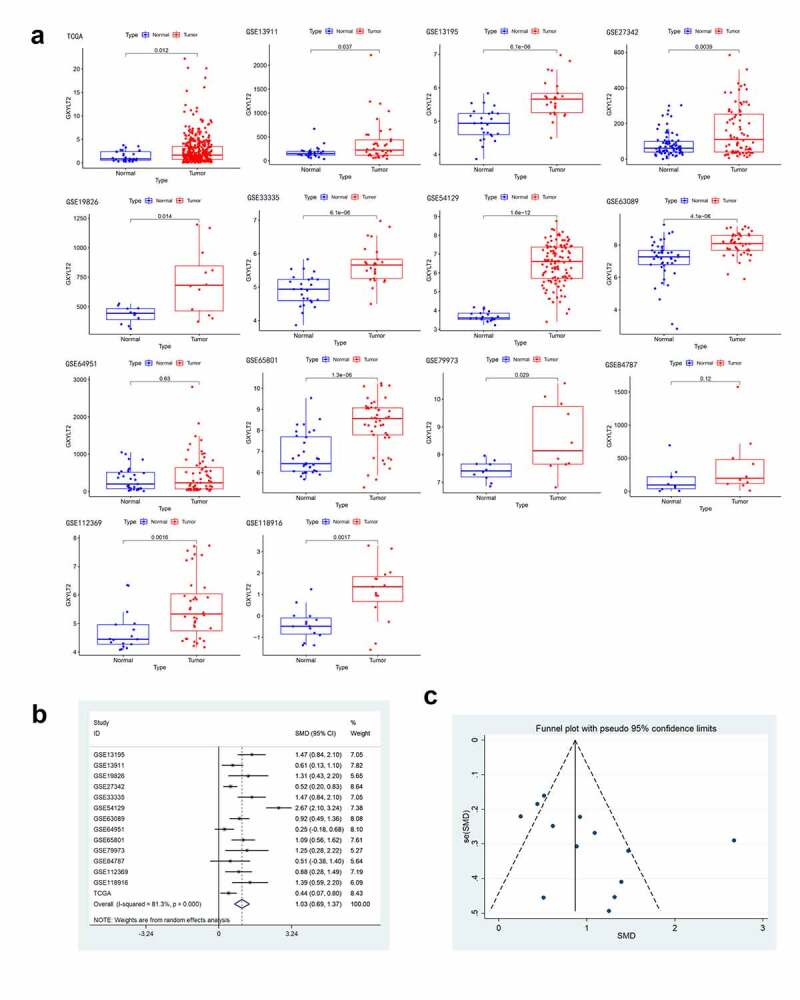
The expression level of GXYLT2 was significantly up-regulated in gastric cancer samples

According to the inclusion and exclusion criteria, 13 datasets were screened from the GEO database (GSE13195, GSE13911 [14], GSE19826 [15], GSE27342 [16], GSE33335 [17], GSE54129, GSE63089 [18], GSE64951 [19], GSE65801 [20], GSE79973 [21], GSE84787 [22], GSE112369 [23], and GSE118916 [24]) in this study. And the expression profile data of GXYLT2 mRNA for GC tissues and controls acquired from TCGA database were also included into the analysis. There were 877 patients with gastric cancer (cases) and 386 individuals without gastric cancer (controls) for the overall analysis (Table 1). In the TCGA database and in 12 GEO datasets (except GSE64951), GXYLT2 was highly expressed in GC tissues (Figure 1(a)). In addition, a meta-analysis of GXYLT2 expression levels in GC was performed. According to the result of the heterogeneity test (*I^2^ *= 81.3%), a random-effects model was selected for this meta-analysis. The results showed that GXYLT2 was upregulated in GC tissues [SMD = 1.03, 95% confidence interval (CI) = 0.69–1.37; P < 0.001] (Figure 1(b)). The symmetrical shape of the funnel plot suggested that there was no evidence of publication bias (Figure 1(c)). And no publication bias was also detected by Begg’s test weighted regression (P = 0.112). Based on these results, GXYLT2 was highly expressed in gastric cancer tissues.

**Table 1.** The main information of GC expression analyses from TCGA data and GEO datasets

Data set	Platform	# GC	# Normal	Country
GSE13195	GPL5175	25	25	China
GSE13911	GPL570	38	31	Italy
GSE19826	GPL570	12	12	China
GSE27342	GPL5175	80	80	USA
GSE33335	GPL5175	25	25	China
GSE79973	GPL570	10	10	China
GSE63089	GPL5175	45	45	China
GSE64951	GPL570	63	31	USA
GSE65801	GPL14550	32	32	China
GSE54129	GPL570	111	21	China
GSE84787	GPL17077	10	10	China
GSE112369	GPL15207	36	17	Japan
GSE118916	GPL15207	15	15	China
TCGA	NR	375	32	USA

(a) GXYLT2 was highly expressed in GC tissues based on TCGA database and GEO database (except GSE64951). The results of forest plot (b) and funnel plot (c) showed no significant publication bias was observed during the meta-analysis of GXYLT2 expression in GC tissues.

### GXYLT2 could be used as a diagnostic marker for patients with gastric cancer

**Figure 2.**
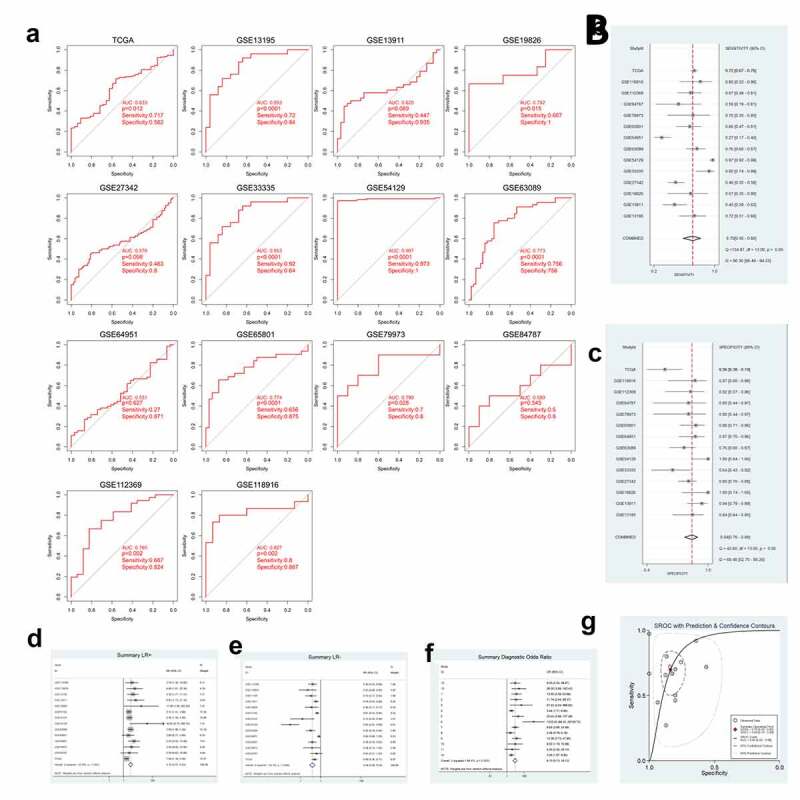
GXYLT2 could be used as a diagnostic marker for patients with gastric cancer based on the results of analysis of the diagnostic values

According to the GXYLT2 expression data from the TCGA data and 13 GEO datasets, ROC curves were plotted (Figure 2(a)). Among them, 9 GEO and TCGA datasets indicated strong diagnostic values (P < 0.05). A further meta-analysis of TCGA data and GEO data was performed on SEN and SPE data from these 14 GXYLT2 expression studies, which were pooled and presented as forest plots. From the sensitivity and specificity data, there was significant heterogeneity between studies (*I^2^ *= 90.36% and *I^2^ *= 69.48%, respectively), therefore a random-effects model was chosen in the analysis of pooled data: SEN, 0.70 (95% CI: 0.56–0.80) (Figure 2(b)); SPE, 0.84 (95% CI: 0.76–0.89) (Figure 2(c)); PLR, 3.10 (95% CI: 2.27–4.23) (Figure 2(d)); NLR, 0.39 (95% CI: 0.28–0.54) (Figure 2(e)); DOR, 9.10 (95% CI: 5.13–16.12) (figure 2(f)); diagnostic accuracy was assessed by plotting the comprehensive receiver operating characteristic curve (SROC) and calculating the AUC (AUC = 0.85, 95% CI: 0.82–0.88) (Figure 2(g)).

(a) Nine GEO datasets and TCGA data indicated that GXYLT2 might have strong diagnostic values for gastric cancer (P < 0.05). Forest plot of sensitivity (b), forest plot of specificity (c), forest plot of positive likelihood ratio (d), forest plot of negative likelihood ratio (e), forest plot of diagnostic odds ratio (f), plot of symmetric summary receiver operating curve (g) of GXYLT2 for GC diagnosis.

### GXYLT2 could be used as a prognostic marker for GC patients

**Figure 3.**
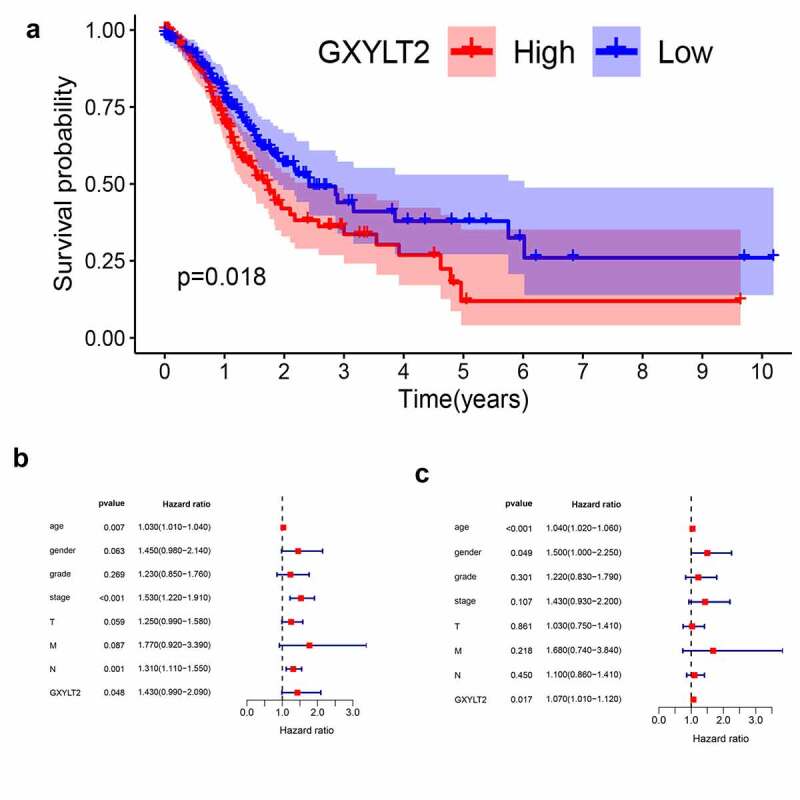
GXYLT2 might serve as a prognostic marker in patients with GC

Based on the TCGA database, all GC samples were split into two groups (high expression group vs low expression group) based on median GXYLT2 expression levels. Relationships between GXYLT2expression and clinicopathological parameters in GC patients were listed in Table 2. The high expression level of GXYLT2 was essentially identified with tumor grade (P = 0.029), pathological stage (P = 0.036), and T classification (P = 0.023). As shown in Figure 3(a), high expression of GXYLT2 was remarkably related to shorter overall survival in both cases (P = 0.018). Univariate Cox regression analysis showed that age (P = 0.007), TNM stage (P < 0.001), lymph node metastasis (P = 0.0011) and GXYLT2 expression (P = 0.048) were significant factors affecting the survival time of patients with GC (Figure 3(b)). Multivariate Cox regression analysis showed that age (P < 0.001), gender (P = 0.049), and GXYLT2 expression (P = 0.017) expression were reliable prognostic indicators in GC patients (Figure 3(c)).

**Table 2.** Relationships between GXYLT2expression and clinicopathological parameters in GC patients

Clinicopathological	GXYLT2 expression		P-value
parameters	High(n = 146)	Low (n = 147)	
Age			
<65	68	58	0.239
≥65	78	89	
Gender			
Female	62	54	0.341
Male	84	93	
Tumor grade			
G1 & G2	44	63	0.029
G3	102	84	
Pathological stage			
I & II	56	79	0.036
III & IV	85	73	
T classification			
T1-T2	29	47	0.023
T3-T4	117	100	
Distant metastasis			
M0	137	136	0.817
M1	9	11	
Lymph node metastasis			
N0-N1	81	87	0.522
N2-N3	65	60

(a) Based on TCGA data, the overall survival of GXYLT2 patients was significantly reduced in the GXYLT2 High expression group (n = 184) compared with the GXYLT2 Low expression group (n = 184). (b) Univariate Cox regression analysis showed that age, TNM stage, lymph node metastasis, and GXYLT2 expression were significant factors affecting the survival time of GC patients. (c) Multivariate Cox regression analysis showed that age, gender, and GXYLT2 expression were reliable prognostic indicators in GC patients.

### GXYLT2 might be associated with multiple tumor and immune regulatory pathways by GSEA

**Figure 4.**
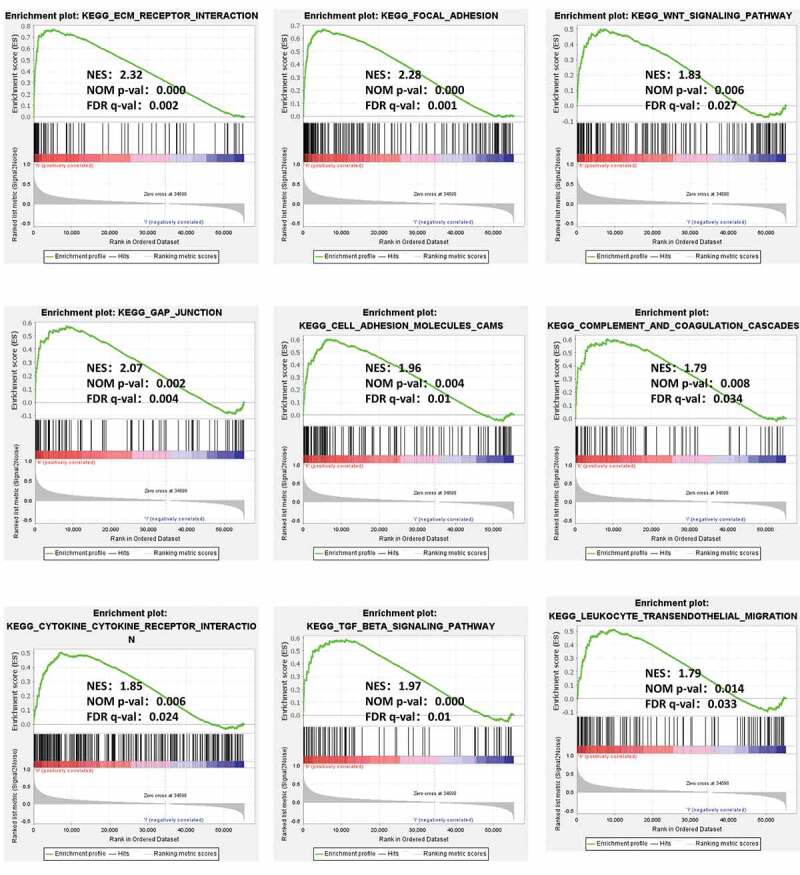
GSEA identified GXYLT2-related tumor pathways and immune regulatory. The terms related to tumor pathways were ‘ECM Receptor Interaction,’ ‘Focal Adhesion,’ ‘Wnt Signaling Pathway,’ ‘Gap Junction,’ and ‘Cell Adhesion Molecules (CAMs)’. The terms related to immunity were ‘Complement and Coagulation Cascades,’ ‘Cytokine Receptor Interaction,’ ‘TGF Beta Signaling Pathway,’ and ‘Leukocyte Transendothelial Migration’

To analyze the potential molecular mechanism of GXYLT2 in gastric carcinogenesis, GSEA was performed on two groups of samples (high expression groups vs low expression groups). Among the 178 enriched pathways, 90 pathways were upregulated, and there were 47 pathways with NOM (nominal) p-value < 0.05. The terms related to tumor pathways were ‘ECM Receptor Interaction,’ ‘Focal Adhesion,’ ‘Wnt Signaling Pathway,’ ‘Gap Junction,’ and ‘Cell Adhesion Molecules (CAMs)’. The terms related to immunity were ‘Complement and Coagulation Cascades,’ ‘Cytokine Receptor Interaction,’ ‘TGF Beta Signaling Pathway,’ and ‘Leukocyte Transendothelial Migration’. The results were shown in Figure 4.

### GXYLT2 was highly correlated with tumor infiltrating immune cells in GC

**Figure 5.**
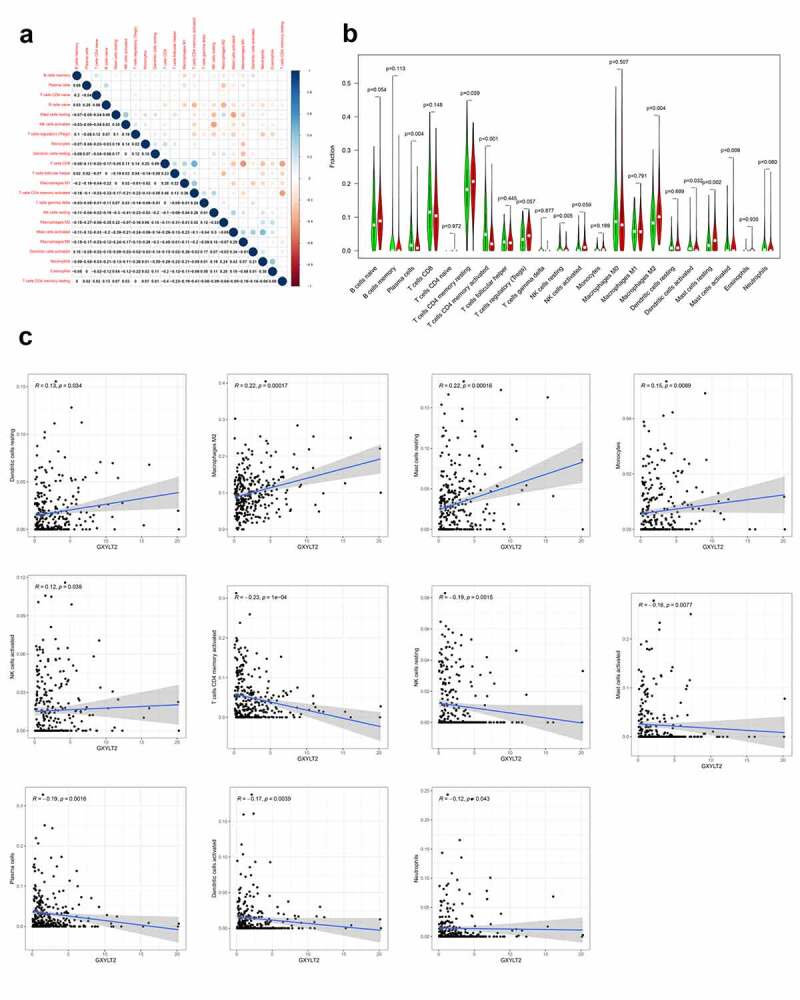
Identification of differences and correlation between the GXYLT2 expression level and tumor-infiltrating immune cells in in GC patients

To explore the correlation of GXYLT2 expression with tumor-infiltrating immune cells, the proportions of immune cell subsets were identified using the CIBERSORT algorithm, and 22 immune cells in gastric cancer samples and the correlation between them were analyzed (Figure 5(a)). The results of the differences and correlation between GXYLT2 expression and tumor-infiltrating immune cells showed that 11 tumor-infiltrating immune cells were significantly correlated with GXYLT2 expression (Figure 5(b)). Among them, resting dendritic cells, M2 macrophages, monocytes, activated NK cells and resting mast cells were positively correlated with GXYLT2 expression. Plasma cells, activated memory CD4 T cells, resting NK cells, activated dendritic cells, and activated neutrophils and mast cells were negatively correlated with GXYLT2 expression (Figure 5(c)).

(a) The correlation analysis demonstrated the relationship between the GXYLT2 expression and immune cells infiltration. The sizes of dots showed the relationship extents. The red dots indicated the positive correlations, and the blue dots indicated the negative correlations. The numbers indicated the coefficients of correlation between genes expression and cells infiltration. (b) Violin plots showed that the proportional differentiation of 22 tumor-infiltrating immune cells in GC specimens with high and low GXYLT2 expression relative to the median GXYLT2 expression was performed using Wilcoxon rank-sum. (c) Eleven tumor immune cells were significantly associated with GXYLT2. The GXYLT2 expression level was correlated positively with five tumor-infiltrating immune cells (resting dendritic cells, m2 macrophages, monocytes, active NK cells and resting mast cells), and was negatively correlated with six tumor-infiltrating immune cells (plasma cells, activated memory CD4 T cells, resting NK cells, activated dendritic cells, and activated neutrophils and mast cells).

### Verification of upregulation of GXYLT2 expression levels in GC by qRT-PCR and immunohistochemistry test

**Figure 6.**
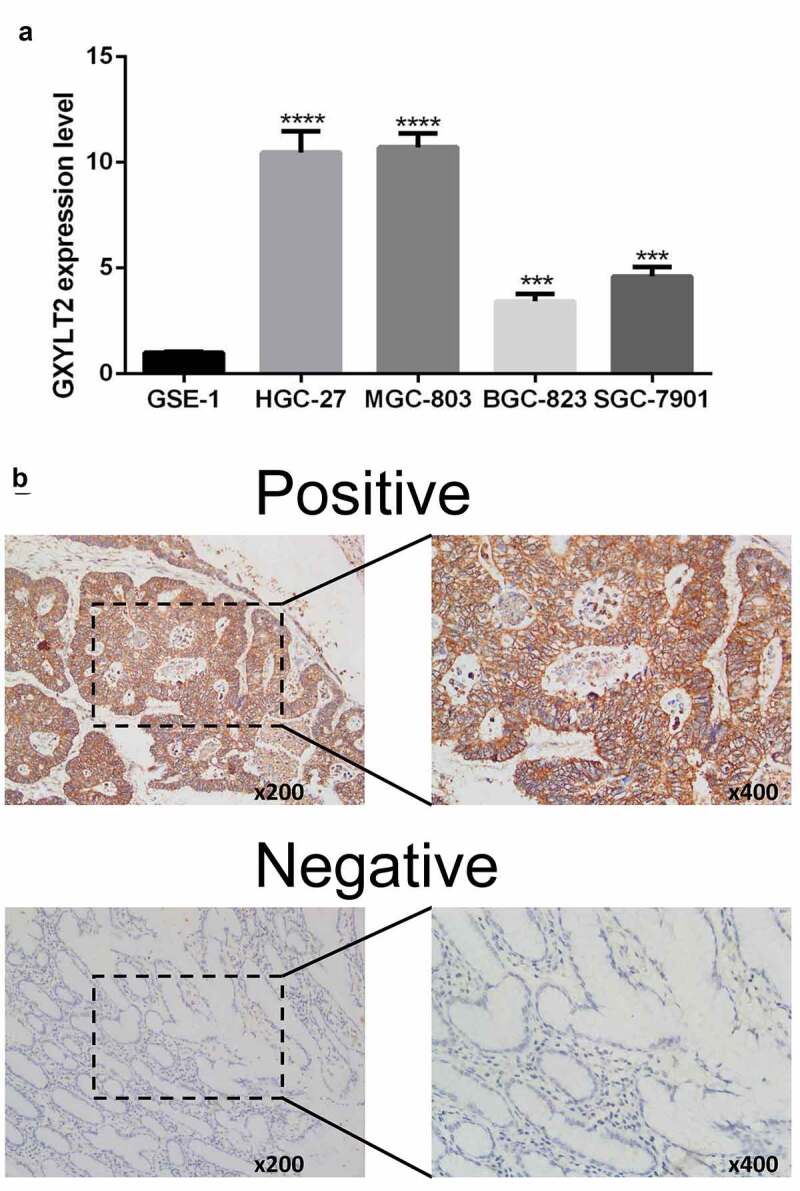
Verification of upregulation of GXYLT2 expression levels in GC by qRT-PCR and Immunohistochemistry test. (a)The mRNA levels of GXYLT2 in normal gastric cell and gastric cancer cell lines. (b) Representative images of GXYLT2 immunohistochemistry in GC tissue

In this study, human gastric epithelial cells (GSE-1) and four GC cell lines (HGC-27, MGC-803, BGC-823, and SGC-7901) were cultured. Compared with that in GSE-1 cells, the expression level of GXYLT2 mRNA in HGC-27, MGC-803, BGC-823 and SGC-7901 cells was significantly higher (P < 0.001) (Figure 6(a)). The results showed that the expression level of the GXYLT2 gene in gastric cancer cells was high, which verified the results of bioinformatics analysis. Immunohistochemical staining results showed that 60% (9/15) of GC tissues had positive GXYLT2 expression. Representative slides were displayed in Figure 6(b). The staining results showed that GXYLT2 was mainly expressed in the cytoplasm and membrane of tumor cells.

## Discussion

GC is a common malignant tumor worldwide. The 5-year survival rate of patients with GC is less than 25% because 80% of patients with GC have reached an advanced stage at the time of diagnosis. Tumor markers used clinically for GC diagnosis include carcinoembryonic antigen (CEA), CA199 (carbohydrate antigen 199), CA125, CA24-2, CA50 and pepsinogen [25]. The diagnostic accuracy of these serum markers is small, and to date, none of these markers are used as the only way to diagnose GC [26]. At present, the mechanism of gastric carcinogenesis is unknown. It is well known that abnormal gene expression might participate in tumorigenesis and can be a diagnostic and prognostic marker for tumors [27]. Recent studies have identified different types of gene biomarkers based on GEO datasets and TCGA data. For example, collagen type I alpha 2 chain (COL1A2) was suggested to be a novel biomarker to enhance the clinical prediction of gastric cancer [28], and collagen type X alpha 1 (COL10A1) might be a key independent predictor of poor over survival of GC patients [29]. The results of the meta-analysis and bioinformatics analysis suggested that FKBP prolyl isomerase 10 (FKBP prolyl isomerase 10, FKBP10) has significant value in the treatment of gastric cancer [30]. In this study, we first confirmed by a meta-analysis that GXYLT2 expression was remarkably higher in GC tissues (SMD = 1.03). Diagnostic meta-analysis results showed that GXYLT2 might be considered a diagnostic marker for gastric cancer and that GXYLT2 expression was remarkably correlated with poor survival prognosis and remarkably negatively correlated with the grade, depth of invasion and TNM stage in patients with GC. The above results show that GXYLT2 may act as an oncogene to enhance GC development.

There are few studies on GXYLT2 in tumors, especially in gastric cancer. Experimental studies in humans *in vitro* showed that GXYLT2 was associated with the occurrence of a variety of tumors [8]. The results showed that GXYLT2 was highly expressed in GC cells and breast cancer cells and that GXYLT2 was mainly localized to the Golgi apparatus. Knock down of GXYLT2 significantly inhibited the proliferation and migration of cancer cells. Further flow cytometry analysis showed that the inhibition of proliferation capacity was correlated with G1/S phase arrest, while high expression of GXYLT2 showed the opposite results. In nude mice tumorigenesis experiments, the tumors formed by GXYLT2-overexpressing cells were smaller than those formed by control cells and knock down of GXYLT2 in breast cancer cells inhibited tumor formation. In GC cells, upon inhibition of GXYLT2, the expression levels of matrix metallopeptidase 2 (MMP2), N-cadherin and vimentin were upregulated, while E-cadherin was downregulated, which was approved by bioinformatics analysis based on multiple data sets [31]. Moreover, the inhibition of GXYLT2 in GC cells resulted in a significant decrease in endogenous Notchintracellular domain (NICD) levels, as well as a significant downregulation of the Notch target gene HES family BHLH transcription factor 1 (Hes1), and high expression of GXYLT2 had the opposite effect. This result suggested that GXYLT2 could promote Notch signaling pathway activation in human cells [7]. In addition, colorectal cancer cells were cultured in acidic media for more than three months. GXYLT2 expression and cancer cell proliferation was decreased in acid-adapted colorectal cancer cells invasion was decreased, and tumor selectivity was high in acidosis compared with neutral healthy tissues [32].

Numerous studies have revealed the importance of the immune microenvironment in tumorigenesis [33]. Tumor-infiltrating immune cells and cytokines are important inhibitors of tumor development [34]. Recent research has shown that surface tumor-infiltrating immune cells are significantly correlated with the prognosis of GC patients, as high levels of tumor-infiltrating lymphocytes are significantly positively correlated with the survival and tumor stage of gastric cancer patients [35]. Immunotherapy has also made great strides in gastric cancer, with pembrolizumab approved for the treatment of patients with recurrent locally advanced or metastatic GC expressing PD-L1 [36]. In this study, the correlation between GXYLT2 expression and tumor-infiltrating immune cells in gastric cancer was explored, and the analysis of the proportion of tumor immune infiltrating cells by CIBERCSORT showed that resting dendritic cells, M2 macrophages, and resting mast cells were positively correlated with GXYLT2 expression. Chen et al. showed that Chitinase-3-like protein 1 (CHI3L1) secreted by M2 macrophages promotes metastasis of gastric and breast cancer cells both *in vitro* and *in vivo* [37]. The immunotherapy results showed that low-dose paclitaxel can inhibit the M2 macrophage phenotype and can induce the M1 phenotype via TLR4 signaling, and clinical doses can kill tumor cells [38]. Therefore, GXYLT2 expression was positively correlated with M2 macrophages, which in turn accelerated the development of gastric cancer, leading to a poorer prognosis.

There are several limitations to this study. Firstly, a significant limitation is the limited number of eligible studies. Although thorough research on public databases and published literature, the specimens from the patients and controls were not very large. More studies with large samples are urgently needed to analyze further the GXYLT2 role in the process of GC pathogenesis. Secondly, the accuracy differences between the public databases (GEO and TCGA) used for data analysis and the choice of statistical methods might affect the interpretation of the results. Thirdly, the lack of raw data from some original studies, such as the exact clinical and surgery data, could imply that a potential interaction between disease factors and GXYLT2 could not be determined. However, the results were verified by analyzing multiple databases, and similar results were obtained by in vitro cell study and Immunohistochemistry analysis.

## Conclusion

In this study, we confirmed that the expression level of GXYLT2 was higher in gastric cancer than in normal gastric tissues. The results demonstrated for the first time that GXYLT2 might play a role during the process of the pathogenesis in gastric cancer via several signaling pathways, and the expression level of GXYLT2 was correlated with infiltrating immune cells. The univariate and multivariate cox regression analysis suggested that GXYLT2 expression might be the independent risk factor for poor survival of GC patients, and GXYLT2 might be a potential prognostic marker in GC patients.

## Data Availability

Publicly available datasets were analyzed in this study, these can be found in GEO database (https://www.ncbi.nlm.nih.gov/geo), and The Cancer Genome Atlas (https://portal.gdc.cancer.gov). The authors confirm that the data supporting the findings of this study are available within the article and its supplementary materials.

## Highlights

(1) GXYLT2 as an oncogene in gastric cancer

(2) High expression of GXYLT2 might be a risk factor for patients with gastric cancer

(3) GXYLT2 might be a potential diagnostic and prognostic biomarker for gastric cancer
